# Insulin Regulates Glut4 Confinement in Plasma Membrane Clusters in Adipose Cells

**DOI:** 10.1371/journal.pone.0057559

**Published:** 2013-03-08

**Authors:** Vladimir A. Lizunov, Karin Stenkula, Aaron Troy, Samuel W. Cushman, Joshua Zimmerberg

**Affiliations:** 1 Program in Physical Biology, Eunice Kennedy Shriver National Institute of Child Health and Human Development, National Institutes of Health, Bethesda, Maryland, United States of America; 2 Experimental Diabetes, Metabolism, and Nutrition Section, Diabetes, Endocrinology, and Obesity Branch, National Institute of Diabetes and Digestive and Kidney Diseases, National Institutes of Health, Bethesda, Maryland, United States of America; Consiglio Nazionale delle Ricerche, Italy

## Abstract

Insulin-stimulated delivery of glucose transporter-4 (GLUT4) to the plasma membrane (PM) is the hallmark of glucose metabolism. In this study we examined insulin’s effects on GLUT4 organization in PM of adipose cells by direct microscopic observation of single monomers tagged with photoswitchable fluorescent protein. In the basal state, after exocytotic delivery only a fraction of GLUT4 is dispersed into the PM as monomers, while most of the GLUT4 stays at the site of fusion and forms elongated clusters (60–240 nm). GLUT4 monomers outside clusters diffuse freely and do not aggregate with other monomers. In contrast, GLUT4 molecule collision with an existing cluster can lead to immediate confinement and association with that cluster. Insulin has three effects: it shifts the fraction of dispersed GLUT4 upon delivery, it augments the dissociation of GLUT4 monomers from clusters ∼3-fold and it decreases the rate of endocytic uptake. All together these three effects of insulin shift most of the PM GLUT4 from clustered to dispersed states. GLUT4 confinement in clusters represents a novel kinetic mechanism for insulin regulation of glucose homeostasis.

## Introduction

With half of the genome devoted to membrane proteins, complex regulatory mechanisms have evolved to govern their activity and location on the plasma membrane of the cell. One of the most critical functions of the membrane is the transport of metabolites into the cell, so one would expect a number of levels of control over transporter activity. To understand this regulation of transporters, it is necessary to obtain both structural and dynamic information on length scales below the diffraction limit. Electron microscopy has ample resolution but lacks optimal kinetics. Super-resolution techniques [1], [2], [3] allow sufficient temporal and lateral resolution to begin to determine the relationship between the functional state of the protein and its mobility in the plasma membrane of living cells (PM) [4], [5].

Because of the clinical importance in type II diabetes of the activity of GLUT4, the glucose transporter expressed primarily in insulin responsive tissues [6], [7], it is one of the best-studied regulatory systems for a transporter. There are excellent recent reviews that summarize up-to-date knowledge about the biochemistry of this regulation [8], [9], [10] and discuss in detail the biogenesis of specialized GLUT4 storage vesicles (GSV) [10], insulin signaling cascades involved in the regulation of GSV exocytosis and GLUT4 translocation to the plasma membrane (PM) [8], [9], and mechanisms of GLUT4 endocytosis, and sorting back to GSV [9], [10]. However, comparatively less is known about dynamics of GLUT4 already present in the PM, where it actually performs its function of facilitating the transport of glucose. The recent finding of GLUT4 clustering suggests that lateral distribution of GLUT4 in the PM is also regulated by insulin and might be important for overall glucose metabolism in adipose [11] and muscle cells [12]. Data from fluorescence recovery after photobleaching studies on GLUT4 diffusion in the PM reveal that PM GLUT4 divides into clustered and freely diffusing fractions; the range of GLUT4 diffusion constants is 0.09–0.14 µm^2^/s for the freely diffusing fraction [13], [14], [15]. Without insulin stimulation (the basal condition), 5–10% of total cellular GLUT4 locates in the PM; most of the total GLUT4 concentrates in GSV [16], [17]. When stimulated by insulin, GSV fuse to the plasma membrane and mostly disperse, increasing the fraction of GLUT4 in the PM and enabling faster glucose uptake by the cells [11], [13], [18], [19]. GLUT4 then undergoes endocytosis (ending its activity in transporting glucose) and traffics to re-sorting endosomes (that package it with other proteins to produce recycled GSV). In our previous work [11] we reported that insulin not only stimulated GLUT4 exocytosis to PM but also affected post-fusion fate of GLUT4 by shifting most of the exocytosis events from “fusion-with retention” to “fusion–with-dispersal” mode. However, the previous study was limited in resolution to the wavelength of light. Since this time, there has been a revolution in optical imaging allowing cell biologists to image single molecules and localize them with spatial uncertainties much smaller than the wavelength of light.

In this report, we use a novel photoswitchable GLUT4 probe with super-resolution total internal reflection fluorescence microscopy (TIRF) to investigate the mechanism of GLUT4 retention in clusters, the molecular dynamics governing GLUT4 exchange in the PM, and the role of insulin in the regulation of these two aforementioned events. We quantify the rate constants of association and dissociation of GLUT4 from PM clusters as well as individual events of GLUT4 delivery by exocytosis and internalization from PM by endocytosis. Based on our data, we propose a model for GLUT4-specific confinement in PM clusters that adds a new mechanism to those existing for regulation of GLUT4 residency in the PM by insulin.

## Results

### HA-GLUT4-EOS Activation Photophysics Allows its Selective Imaging on PM

To study GLUT4 localization and dynamics simultaneously, we constructed a plasmid for a fusion protein of GLUT4 with both an extracellular HA tag and an intracellular photo-switchable fluorescent tag (EOS). The correct localization of HA-GLUT4-EOS was verified by immunostaining with HA and GLUT4 antibodies (Fig. S1a). Isolated adipose cells transfected with HA-GLUT4-EOS typically showed a ∼3-fold increase in cell surface HA-antibody binding after insulin-stimulation (Fig. S1b). While this value is low compared to estimates of in-situ increases in endogenous GLUT4, this diminished response is probably due to a number of reasons including isolation from tissue, electroporation and the overnight culturing necessary to achieve detectable levels of exogenous protein expression. However, the same level of response is typical for another plasmids, HA-GLUT4 and HA-GLUT4-GFP in similar settings [20].

**Figure 1:**
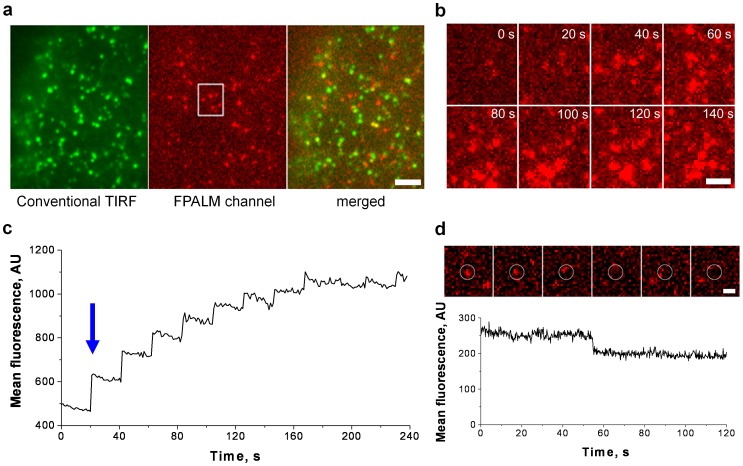
Photo-Activation of GLUT4-EOS in the Plasma Membrane Clusters. (**a**) Simultaneous TIRF/FPALM imaging of GLUT4-EOS. Non-activated GLUT4-EOS is detected in the green channel and shows abundant plasma membrane clusters. Individual activated GLUT4-EOS molecules are detected in the red channel. Bar, 5 µm. See also Movie S1 (**b**) Zoomed region from (A) depicting the sequential activation of a GLUT4-EOS. Bar, 2 µm. (**c**) A plot of mean fluorescence of activated GLUT4-EOS from region shown in (**b**) (**d**) Series of frames depicting single GLUT4-EOS molecule photo-bleaching event (upper panel); lower panel shows corresponding step-wise drop in fluorescence intensity measured within circular region. Bar, 1 µm.

UV irradiation of the expressed protein induced the expected shift from green to red fluorescence (fig. 1a–c). Thus, by controlling UV exposure, we activate and monitor a small fraction of expressed HA-GLUT4-EOS in the red emission channel (561 nm excitation laser), while the non-activated HA-GLUT4-EOS is detected at 488 nm (fig. 1a). Non-activated EOS was used to monitor vesicular trafficking using conventional TIRF, while sparse activation of EOS allowed single particle tracking of individual GLUT4 in the plasma membrane (Movie S1).

Adipose cells were isolated as primary cells from epididymal fat pad tissue of Sprauge Dawley (180–200 g) rats, and were transfected with HA-GLUT4-EOS and imaged 18–24 h later. In a 4 min time-course, a series of 50 ms pulses of 405 nm laser every 20 s showed how each pulse increased the number of activated HA-GLUT4-EOS molecules (fig. 1b), measured as a step-wise increase of the integrated fluorescence signal in the red channel (fig. 1c). With sparse activation of GLUT4-EOS molecules (∼ 0.01–0.1 activated EOS/µm^2^), there was robust detection of single molecules over ∼30–60 s. Bleaching of single molecules was detected as a step-wise drop of fluorescence (fig. 1d).

There was a gradual saturation of activated HA-GLUT4-EOS signal in cells illuminated with 10–15 consecutive UV pulses (fig. 1b), as the available GLUT4-EOS molecules were converted into the red state. We could selectively activate HA-GLUT4-EOS in the PM (TIRF), or internal to GSV, endosomes, and other organelles (wide-field illumination). The probability of photoactivation in TIRF decreased with distance from the interface much faster than the intensity of the evanescent wave. While with conventional TIRF (at 488 nm), with a penetration depth of ∼100 nm, the difference in excitation probability of HA-GLUT4-EOS present at the interface and at 100 nm was about 3-fold, for FPALM activation (at 405 nm) the probability of photoconversion for HA-GLUT4-EOS molecules localized in the same intracellular structures 100 nm away from the PM dropped more than 12-fold compared to HA-GLUT4-EOS present in the PM (data not shown). This effect can only partially be explained by the dependence of TIRF penetration depth on laser wavelength. Indeed, for the same incident angles, exponential decay of an evanescent wave of *λ* = 405 nm and *λ* = 488 nm at *z = δ(488)* can be calculated as follows: δ(λ)∼λ; I_λ_(z) = I_0_*exp(- z/δ(λ)); I_488_(z)/I_405_(z)|_z = δ(488)_ = exp(1- λ_405_/λ_488_)∼1.6. We found this effect to be beneficial for selective activation of HA-GLUT4-EOS at the PM compared to intracellular sites.

### Single Molecule Imaging: Tracking GLUT4 Diffusion in the PM

**Figure 2:**
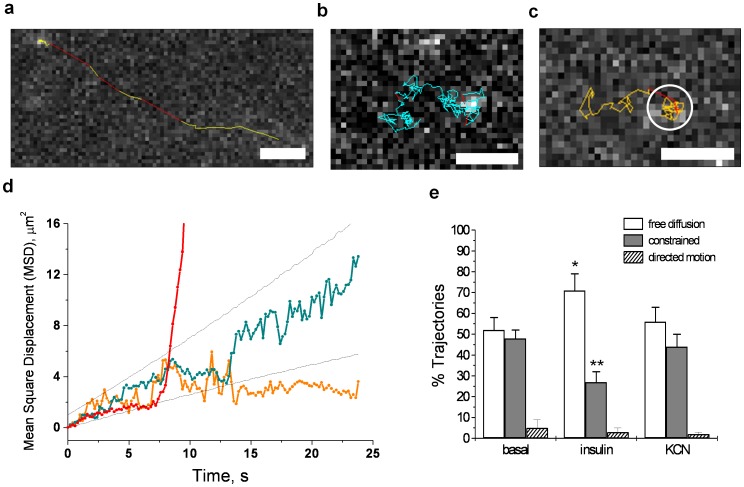
Tracking GLUT4-EOS Molecules. Trajectories of GLUT4-EOS molecules exhibiting different types of motion: (**a**) directed motion corresponding to vesicular transport (see Movie S2)**;** (**b**) free lateral diffusion (see Movie S3); (**c**) constrained diffusion within plasma membrane cluster (see Movie S4). All bars, 2 µm. (**d**) Graphs of the Mean Square Displacement (MSD) of GLUT4-EOS molecules for three distinct types of motion: directed movement (red), free lateral diffusion (blue), and constrained diffusion within a cluster (orange). Dashed lines correspond to 95% confidence intervals obtained from simulation. (**e**) Percentage of trajectories categorized into three types of motion observed, based on the analysis of MSD. Trajectories containing at least 30 time points were scored as directed motion or constrained diffusion if five or more points were above or below 95% confidence interval bounds. At least 150 trajectories were analyzed for each condition. Error bars are SEM, N = 15 cells. *p<0.05; **p<0.01.

Individual fluorophore positions of HA-GLUT4-EOS were localized at intervals of 200 ms at spatial uncertainties of less than 30 nm over times of 30–60 seconds (fig. 2a–c). In order to distinguish individual molecules in diffraction-limited images, the average distance between activated GLUT4-EOS has to be at least 250 nm. However, to do a robust tracking of mobile molecules for several frames, it is also important that the trajectories of individual molecules do not intersect for the given interval. This was achieved in practice when the density of activated molecules was below 0.2 per µm^2^. In our experiments we adjusted the duration of the 405 nm activating pulse to keep the density of activated GLUT4-EOS molecules between 0.01–0.1 per µm^2^.

We analyzed 162 trajectories in 15 basal cells and 196 trajectories in 15 insulin-stimulated cells from six independent experiments and observed three major types of motion patterns: directed motion (fig. 2a), random diffusion (fig. 2b), and confined diffusion (fig. 2c).

Directed motion, the least common pattern, was observed when GLUT4-EOS was activated in the wide-field photoactivation mode for a 405 nm laser (fig. 2a). When GLUT4-EOS was activated in the TIRF mode, less than 5% of trajectories exhibited this directed motion. Further, in cells co-expressing GLUT4-EOS and tubulin-GFP, directed motion of GLUT4-EOS was always associated with intracellular GLUT4-containing vesicles moving along microtubules (Movie S2). Vesicles labeled with HA-GLUT4-EOS showed the same higher trafficking in the basal state and the same lower trafficking in the insulin-stimulated state that were observed previously with HA-GLUT4-GFP. Moreover, in control experiments, when both constructs were expressed, the directed motion of activated HA-GLUT4-EOS was confirmed to co-localize with the motion of GLUT4-GFP labeled structures. Thus, the directed motion of activated HA-GLUT4-EOS was attributed to movement of intracellular organelles, and was excluded from analysis of PM GLUT4.

When HA-GLUT4-EOS was activated selectively in the PM by using TIRF photo-conversion, most of the GLUT4-EOS molecules exhibited only random diffusion (fig. 2b, Movie S3) or confined motion (fig. 2c, e, Movie S4). A molecule exhibiting random walk motion was typically tracked for 10–30 seconds until the molecule photobleached or its trajectory overlapped with that of another molecule. These trajectories were analyzed both individually and as pooled data to measure average diffusion coefficients. Trajectories that exhibited significant deviation of MSD from linear behavior (fig. 2d) were segregated for separate analysis and excluded from the population of randomly diffusing GLUT4. The estimated diffusion coefficient for molecules undergoing free diffusion (D_avg_ = 0.092±0.008 µm^2^/s) was found to be in good agreement with previous reports (0.093–0.14 µm^2^/s) using two very different methods, FRAP [13] or the diffusion of GLUT4 away from the site of exocytosis [15]. The random walk motion of individual HA-GLUT4-EOS was observed over the whole area of the PM, and unlike some other proteins [4], [5], there were no regions where GLUT4 was excluded from entering.

However, a significant fraction of the HA-GLUT4-EOS activated in the PM exhibited constrained diffusion (fig. 2c, e) that colocalized with bright puncta of GLUT4 clusters visible in green channel (Fig.1a). In the basal state, 48±4% of trajectories were classified as constrained diffusion, and 52±6% of trajectories were defined as free diffusion (±SEM, N = 15 cells). Insulin stimulation increased relative fraction of molecules undergoing free diffusion 71±7% (p<0.05), and decreased constrained diffusion 27±5% (p<0.01). To independently estimate the fraction of molecules undergoing constrained diffusion and associated with clusters, we analyzed time-averaged projections of our recordings and compared the integrated intensity of clusters and intensity of uniform background resulting from averaging of multiple diffusing monomers. Consistent with previous measurements, the amount of PM GLUT4 associated with clusters in the basal state was estimated to be 40–50%. Insulin stimulation resulted in increased number of freely-diffusing GLUT4 molecules, and effectively reduced the fraction of GLUT4 confined in clusters to 25–30% (fig. 2e).

To test whether GLUT4 diffusion and confinement was energy-dependent process, we depleted ATP by treating cells with KCN for 15 min [21], [22]. Consistent with previous observations, KCN treatment successfully blocked all microtubule-dependent traffic of GSV and practically eliminated instances of directed motion detected for GLUT4 molecules. However, ATP depletion showed no effect on characteristics of GLUT4 diffusion in PM, and did not change the relative abundance of constrained or freely diffusing molecules.

### Characterization of GLUT4 at the PM Clusters by FPALM and EM

**Figure 3:**
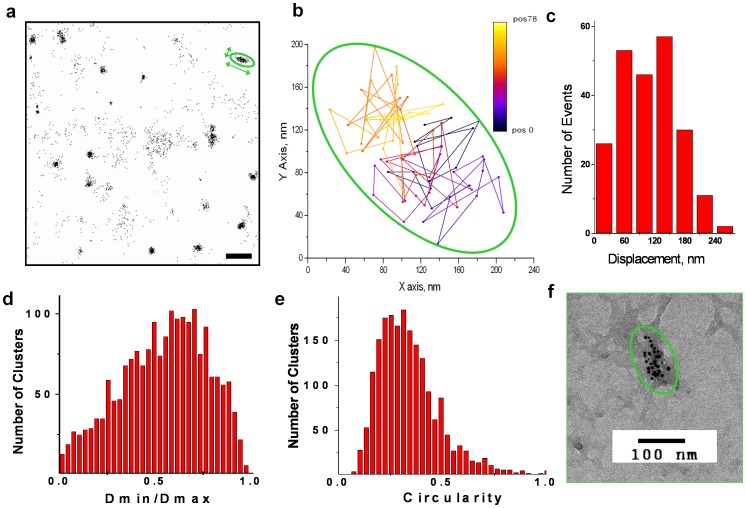
Elongated Shape of GLUT4 Clusters. (a) FPALM reconstruction of GLUT4-EOS positions detected during 600 frames. Clustered positions were associated with GLUT4 molecules trapped inside the clusters. Bar, 1 µm. (b) An example of GLUT4 trajectory within the cluster; sequential positions where GLUT4 molecule was localized are shown in pseudocolor; the bounding ellipse is shown in green. Behavior of GLUT4 within the clusters was characterized by step size distribution (c); shape of clusters was analyzed by (d) fitting with ellipses (Dmin, Dmax – diameters along minor and major axis), and (e) by calculating circularity as 4pi*(area/perimeterˆ2). (f) EM micrograph depicting immuno-gold labeling of plasma membrane with rabbit anti-GLUT4 and 5 nm gold-conjugated goat-anti-rabbit antibodies.

To understand the nature of the forces governing clustering of GLUT4 we performed a series of experiments aimed at measuring the shape of the cluster and the movement of GLUT4 within the cluster. First, we analyzed the motion of individual HA-GLUT4-EOS at the sites of GLUT4 clusters (as defined above). Most of these molecules displayed confined diffusion (fig. 2c and 3b) distinct from the random walk diffusion discussed above. Corresponding MSD curves had significant deviation from linearity and quickly saturated at a certain value significantly below the estimated displacement for random diffusion: MSD(t) <<4Dt (fig. 2d). Typically the size of the confinement zone was within the diffraction limit, and was estimated from the amplitude of MSD fluctuations within the cluster (120±40 nm, N = 225) (fig. 3c). However, the fact that GLUT4 molecules remain mobile within the cluster indicates that simple models of a cross-linked or solid phase domain cannot explain the mechanism of cluster formation.

Further, FPALM images showed that clusters significantly deviated from circular shape. We used FPALM to reconstruct all positions of molecules within the clusters where GLUT4-EOS was detected for at least 30 consecutive frames (fig. 3a and 3b). In total, we analyzed 1960 clusters from 56 basal and 42 insulin-stimulated cells. In these reconstructed images, cluster regions were either fit with ellipses to measure diameters along major and minor axis (fig. 3d), or their circularity was calculated as a ratio of area over perimeter square (fig. 3e) (πA/P^2^, where A is the area of the cluster, and P is the perimeter). While these estimates of circularity and the ratio of the diameters both indicated an elongated shape for the majority of clusters, there were no statistically significant differences between basal and insulin-stimulated conditions. The diameters of the fit ellipses (corrected for the uncertainty of position localization, 30 nm) were 170±30 nm for the major axis and 90±30 nm for minor axis. The average ratio of the diameters was 0.54±0.22 (±SD), while the average circularity was 0.34±0.15 (±SD). The fact that GLUT4 clusters are not circular suggests that the line tension does not play significant role in formation of the clusters; line tension is too low to induce lipid phase demixing as seen in liquid-ordered and liquid-disordered domains. In control experiments, mild depletion of cholesterol using 0.1–1 mM methyl-beta-cyclodextrin (*MBCD*) did not result in any statistically significant changes in cluster shape or cluster density (Fig. S2).

To independently assess the shape and size of GLUT4 clusters, we used an *en-face* technique of immunoelectron microscopy for HA-GLUT4 in the PM of adipose cells [23]. Transmission electron micrographs demonstrated that immunogold-labeled HA-GLUT4 was indeed present in PM clusters as well as in monomers. The number of gold molecules associated with each cluster ranged from 3–20, (fig. 3f), and the average size of the GLUT4 clusters (110±20 nm, N = 36) determined by EM was in agreement with values obtained from MSD analysis (120±40 nm) and FPALM measurements (90–170 nm).

### Association and Dissociation of GLUT4 at the PM Clusters

**Figure 4:**
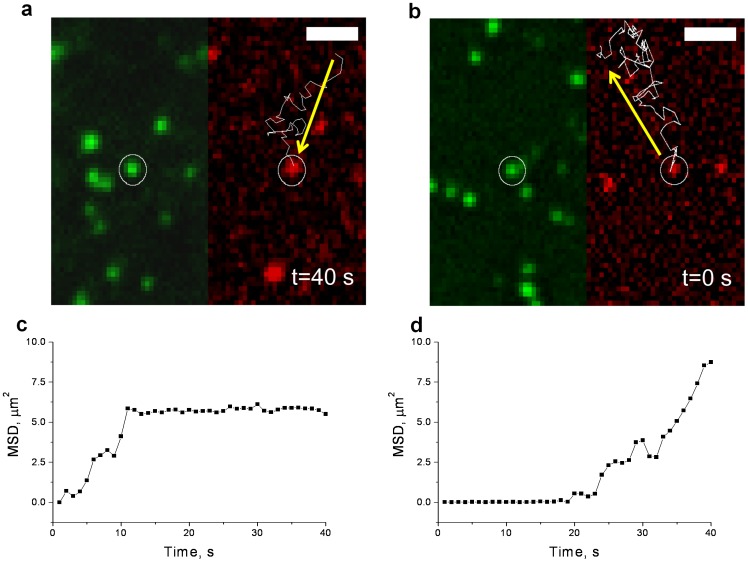
GLUT4 Interaction with Pre-existing Clusters. GLUT4-EOS association with (a) (see also Movie S5) and dissociation from (b) (see also Movie S6) pre-existing GLUT4 clusters. Activated GLUT4-EOS molecule, detected in the red channel, colocalizes with the cluster visible in green channel (non-activated GLUT4-EOS). The last frame is shown for an association event (a), and the first frame is shown for dissociation event (b). Trajectory of the activated GLUT4-EOS molecule is shown in white; white circles depict the site of the cluster. All bars, 2 µm. (c) and (d) show graphs of the Mean Square Displacement (MSD) for association and dissociation events depicted in (a) and (b) correspondingly.

We next attempted to link the structural characteristics of the GLUT4 clusters with their dynamic properties by studying the molecular motion of individual GLUT4 monomers as they associated or dissociated from these clusters. Using conventional techniques in our system, including TIRF, it was impossible to detect the association and dissociation of monomers due to noise. However using FPALM and GLUT4-EOS probes we were able to detect events when activated GLUT4-EOS molecules undergoing random walk motion got trapped and confined at GLUT4 clusters (fig. 4a,c and Movie S5). We also detected events when molecules confined at PM clusters were occasionally released, moving away via random walk motion (fig. 4b, d and Movie S6).

To analyze the GLUT4 lifetime in clusters, we identified regions of PM clusters of non-activated GLUT4-EOS stationary for at least 30 to 60 frames. We then photoswitched an average of one GLUT4-EOS molecule per cluster, and followed their intensity to determine their lifetimes before disappearing from the clusters. We attributed eventual disappearance to three primary factors: photobleaching, dissociation from the clusters, and endocytosis.

**Figure 5:**
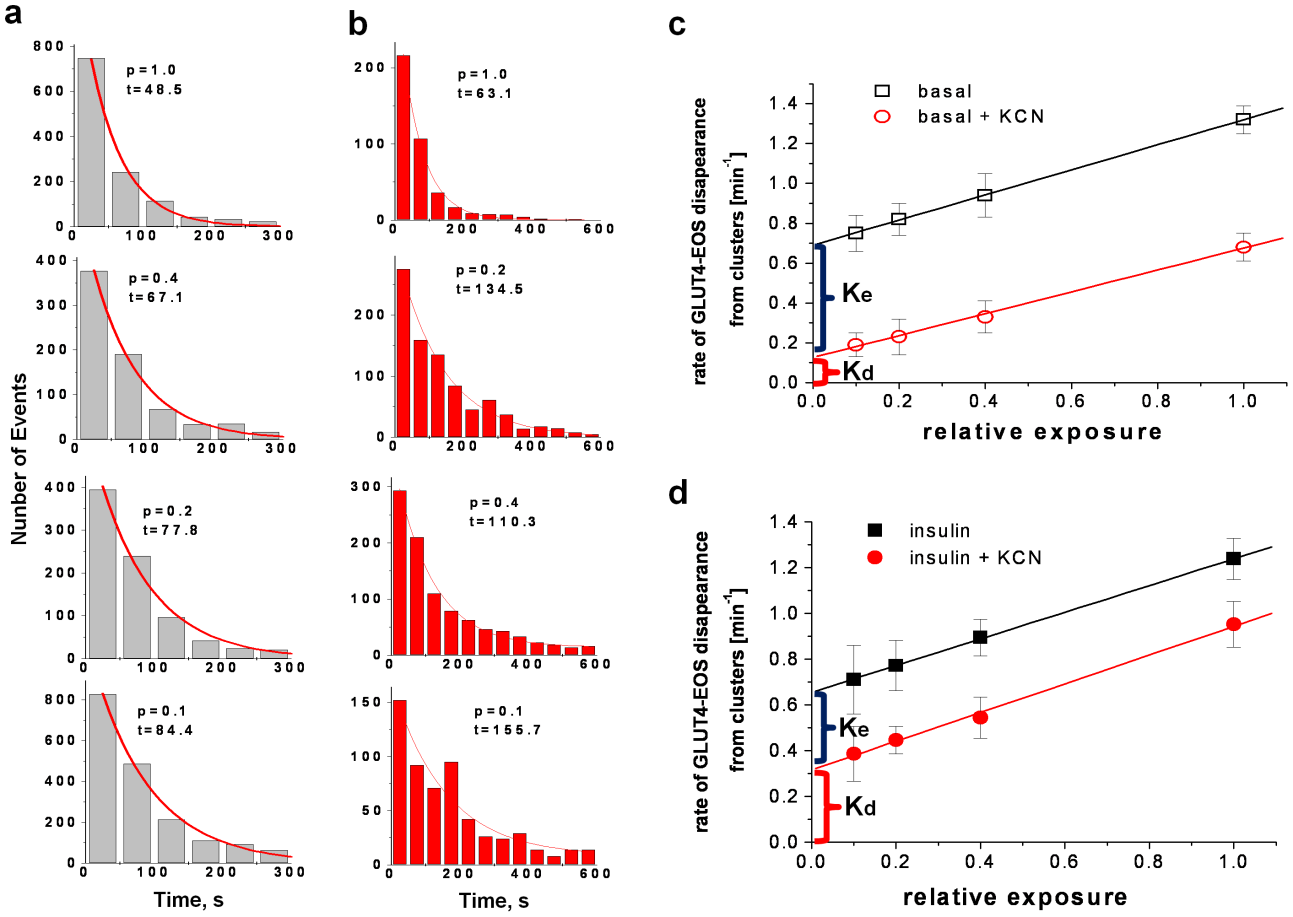
GLUT4 Dwell Time at the Clusters: Rates of Dissociation and Endocytosis. (**a**) and (**b**) - histograms of dwell time for individual GLUT4-EOS molecules localized within the clusters in control cells (gray) and cells treated with KCN (red). Fat cells treated with 2 mM KCN for 15 min showed complete inhibition of GLUT4 endocytosis due to ATP depletion (Kono, Robinson et al. 1977; Quon, Guerre-Millo et al. 1994). Dwell time data was pooled from at least 15 cells for each condition. The dwell time of GLUT4-EOS molecule was measured as the time between appearance or activation of GLUT4-EOS at the cluster site and the time when the molecule was lost for more than three consecutive frames. Exponential fit of the histogram data was used to calculate the rates of disappearance under different illumination protocols for at least 5 cells in each condition (basal, insulin, with and without KCN). The combined rate of disappearance was considered to be a sum of three independent processes (bleaching, lateral dissociation from cluster and endocytosis): K(p) = p*K_b_+K_d_+K_e_, where K_b_ – rate constant of bleaching, p – relative exposure to excitation light, K_d_ and K_e_– rate constants for dissociation and endocytosis from clusters respectively. To control for bleaching, we measured the rates of disappearance at four different illumination protocols with constant exposure t_exp_ = 200 ms but with different intervals between acquisitions (t_int_ = 0.2, 0.5, 1.0, and 2 sec), which correspond to the relative exposure to excitation light p = t_exp_/t_int_ : 1.0; 0.4; 0.2; and 0.1. (**c**) and (**d**) show graphs of combined rate of disappearance K(p) as a function of the relative exposure for basal and insulin stimulated conditions. Red circles correspond to cells pre-treated with KCN for 15 min. The combined rate of disappearance for KCN-treated cells was assumed to be K(p) = p*K_b_+K_d_, with the rate constant of endocytosis (K_e_) being essentially zero. The rate constants of dissociation K_d_ and endocytosis K_e_ from clusters were determined from intersection of linear fit of the data with ordinate axis.

We further attempted to determine the dependence of the rates of disappearance on each of these parameters. The dwell-times of individual GLUT4-EOS molecules within clusters were measured and plotted as a histogram (fig. 5a,b); an exponential fit of these data yields the combined rate of molecular disappearance in all three ways (K(p) = p*K_b_+K_d_+K_e_, where K_b_ – rate of bleaching, p – relative exposure to excitation light, K_d_ – rate of dissociation from clusters; K_e_ – rate of endocytosis;). To exclude the contribution of bleaching, we measured the rates of disappearance at a constant exposure t_exp_ = 200 ms, but with different intervals between acquisitions (t_int_ = 0.2, 0.5, 1.0, and 2 sec). This protocol effectively decreased the relative exposure as p = t_exp_/t_int_ to corresponding values of p = 1.0; 0.4; 0.2; and 0.1. By plotting K(p) as a function of the relative exposure we obtained K_d_+K_e_ by linear extrapolation (fig. 5c,d). To separate the rate of endocytosis and that of dissociation we depleted ATP by treating cells with KCN for 15 min [21], [22]. Consistent with previous experiments, KCN successfully blocked GLUT4 endocytosis and exocytosis [11], but did not affect the distribution of GLUT4 between clusters and individually diffusing monomers (fig. 2e). Using the same linear extrapolation as above, we determined the rate of dissociation from the clusters in basal cells to be 0.12±0.01 min^−1^ (fig. 5c) and in insulin-stimulated cells to be 0.31±0.02 min^−1^ (fig. 5d, mean ±SEM, N = 15 cells, p<0.01). Subtracting this value (K_d_) from the combined rate of endocytosis and dissociation (K_d_+K_e_), we estimated the rate constants for GLUT4 endocytosis from clusters: K_e_ = 0.57±0.07 min^−1^ for basal (fig. 5c) and 0.34±0.05 min^−1^ for insulin stimulated cells (fig. 5d, mean ±SEM, N = 15 cells, p<0.05).

Since KCN did not significantly change the distribution of PM GLUT4 between clustered and non-clustered states, it is logical to assume that the energy of the exchange between the clusters and monomeric GLUT4 in the PM is close to equilibrium. Based on these assumptions, we further estimated the rate constant for GLUT4 association with clusters from the equilibrium equations: K_a_ = K_d_*[free PM GLUT4 ]/[GLUT4 constrained in clusters]: 0.1±0.03 min^−1^ for basal (fig. 5c) and 0.08±0.04 min^−1^ for insulin stimulated cells (mean ±SEM, N = 15 cells, p = 0.7).

### GLUT4 Exocytosis and Cluster Formation

**Figure 6:**
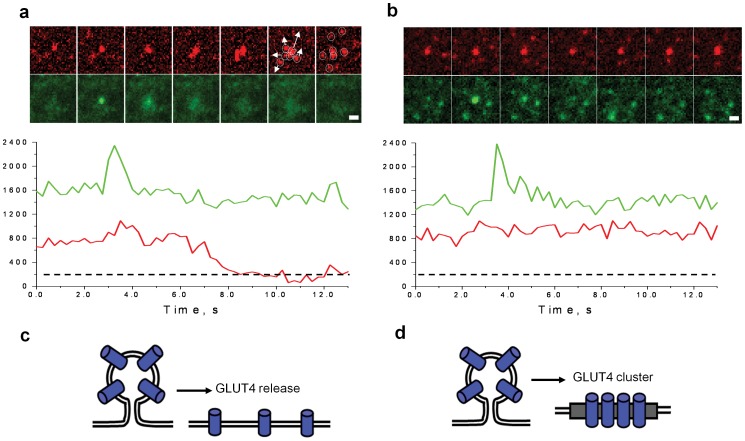
Formation of the Clusters via GLUT4-Specific Retention During Exocytosis. Sequences of consecutive time-lapse frames showing examples of GLUT4 vesicle fusion resulting in complete dispersal of GLUT4 molecules “fusion-with-dispersal” (a) (see also Movie S7) and formation of GLUT4 cluster “fusion-with-retention” (b) (see Movie S8). Lower panels show corresponding graphs for time-lapse fluorescence changes at the site of fusion. Mean fluorescence intensity was calculated for circular regions of 1 µm radius. Red channel corresponds to activated GLUT4-EOS molecules; green channel corresponds to IRAP-pHluorin. IRAP-pHluorin fluorescence spikes correspond to luminal pH equilibration upon fusion pore opening (black arrows). Note that IRAP-pHluorin leaves the site of fusion when GLUT4 forms a cluster. Frames are shown immediately before and after fusion with 200 ms interval. All bars, 1 µm. (c) and (d) show cartoon depictions of GLUT4 molecules leaving site of fusion during “fusion-with-dispersal” and forming a cluster during “fusion-with-retention”.

To investigate the mechanism of GLUT4 cluster formation we monitored the exocytosis of individual GLUT4 vesicles using a combination of HA-GLUT4-EOS and IRAP-pHluorin probes (fig. 6). We co-transfected cells with 2 µg/ml HA-GLUT4-EOS and 8 µg/ml IRAP-PHluorin, a pH sensitive protein whose quantum efficiency is a function of its ambient pH. De-acidification of the GLUT4 storage vesicle upon the opening of the fusion pore results in a rapid increase of the fluorescence intensity of the PHluorin within the lumen of the vesicle that rapidly diminishes as the probe diffuses away from the site of exocytosis, giving a highly detectable “flash” at the instant of fusion [15].

Cells were illuminated with 15–20 consecutive pulses of 405 nm laser light at a wide-field illumination mode, until saturation of the fluorescence intensity signal in the red channel was achieved (fig. 1c). Next, HA-GLUT4-EOS molecules that were present in the PM were bleached for 60 s using TIRF illumination with a 561 nm laser. This protocol resulted in activation of most of the HA-GLUT4-EOS molecules present in GSV and intracellular structures, while minimizing the density of activated HA-GLUT4-EOS in the PM and minimizing bleaching of the HA-GLUT4-EOS in GSV (fig. 1a).

Consistent with previous results (obtained using conventional TIRF microscopy), we detected two types of fusion: “fusion-with-dispersal” (fig. 6a, Movie S7) and “fusion-with-retention” (fig. 6b, Movie S8) [11]. In fusion with dispersal, GLUT4-EOS release coincided with the release of IRAP-PHluorin, or was shortly delayed (0.5–1.5 s), but resulted in complete dispersal of activated HA-GLUT4-EOS contained in the vesicle (fig. 6a).

In fusion with retention, IRAP was released completely, but HA-GLUT4-EOS was retained at the site of fusion where new GLUT4 clusters were being formed (fig. 6b). The mobility of HA-GLUT4-EOS molecules within these newly formed clusters was indistinguishable from those within the other clusters present in the PM.

We never saw de-novo association of the randomly diffusing GLUT4 despite frequent overlap of freely diffusing GLUT4-EOS molecules (∼0.01–0.1 per um^2^ per min depending on the density of activated molecules). This observation is consistent with our previously published data suggesting that clusters are formed as the result of fusion-with-retention. This retention in clusters is specific to GLUT4, as IRAP always leaves the fusion site, independent of GLUT4 retention or release.

In control experiments, we tested the effects of actin-disrupting drugs on the stability of GLUT4 clusters. Treatment of basal cells or insulin–stimulated cells with either cytochalasin D (1 uM) or latrunculin A (1 uM) did not produce a significant change in GLUT4 cluster number as measured using HA-antibody (Fig. S3). However, pretreatment of cells with latrunculin A (1 uM, t = 30 min) before insulin stimulation severely inhibited exocytosis of GSV, which was observed by disappearance of Irap-pHluorin flashes as well as by disappearance of insulin–stimulated increase in PM GLUT4. These data do not rule out the importance of actin in the formation of clusters during exocytosis, but do show that stability of existing GLUT4 clusters is independent of actin.

## Discussion

We have investigated the molecular dynamics that govern the insulin-stimulated increase in glucose transport, as observed through GLUT4 delivery, migration, clustering, and endocytosis. In this report we introduced a photoswitchable HA-GLUT4-EOS probe that allowed us to use conventional fluorescent microscopy simultaneously with super-resolution localization microscopy. Thus we could follow vesicular traffic, GLUT4 exocytosis and endocytosis, as well as track and analyze the behavior of individual GLUT4 molecules in the PM. Our single molecule data indicate that the surface distribution of GLUT4 monomers is governed by two-dimensional random diffusion, while clusters are nucleated by selective confinement of GLUT4 delivered by exocytosis of GSV. GLUT4 molecules diffuse and interact with GLUT4 clusters resident in the PM, but monomers do not interact with each other.

Insulin augments GLUT4 dissociation from clusters into PM and decreases the rate of endocytic uptake thus shifting PM GLUT4 towards dispersed monomers. These effects of insulin are synergetic with the increased delivery of monomers by exocytosis and altogether function to prolong GLUT4 retention in the PM and maximize efficiency of glucose transport. Thus, GLUT4 clusters function as insulin-regulated sites of GLUT4 confinement in the PM and serve an important role in the overall recycling of GLUT4.

### GLUT4 Diffusion and Single Molecule Imaging

To our knowledge, only two studies have followed GLUT4 dynamics at a single molecule level [24], [25]. They used myc-GLUT4 labeled with Quantum dots to track GLUT4 molecules throughout the cell. They observed a significantly wider range of diffusion coefficients (D = 0.00001–1 µm^2^/s) than was reported in the literature for average diffusion coefficients (D_avg_ = 0.09–0.14 µm^2^/s) [13], [15] and observed in our studies for individual molecules (D_ind_ = 0.07–0.16 µm^2^/s). It is important to note that their wide range includes motion of not just PM GLUT4, but also the motion of varied intracellular structures, like jiggling tethered vesicles and moving GSV on the microtubules. Since the different types of motion were not distinguished in these studies, and the MSD was used to quantify the mobility of the overall GLUT4 population, a direct comparison of diffusion coefficients is not useful. The only way to find accurate coefficients is to separate the different types of motion and analyze them separately, as reported here.

While the free-diffusion of individual GLUT4 molecules was observed over the whole area of the PM, the confined motion was restricted to subdiffraction-limited clusters detectable with conventional TIRF. We observed these patterns of free and constrained diffusion in both insulin stimulated and basal conditions. Similar to transient confinement zones reported for glycosylphosphatidylinositol-anchored receptors [26], and some other membrane-associated and transmembrane proteins [5], GLUT4 was observed to switch from free to confined diffusion upon association with clusters. However, the time GLUT4 spent in the clusters (dozens of seconds) was significantly larger than the average confinement time (hundreds of milliseconds) reported for picket-fence models [27].

### Mechanisms of GLUT4 Cluster Formation

In our study GSV fusion with retention leads directly to formation of GLUT4 clusters in the PM, followed by exchange of GLUT4 with the pool of mobile monomers in the PM. The cluster may have been pre-formed in the GSV or created upon fusion. If the particular chemistry of the PM does not determine the post-fusion fate of GLUT4, it is likely that intermolecular interactions already in place in particular GSV suffice.

We did not observe spontaneous association of GLUT4-EOS molecules in the absence of pre-existing clusters, indicating that interaction of GLUT4 monomers is not enough for cluster formation. Rather, the presence of an additional protein entity or specific lipid environment is needed to cluster GLUT4 molecules.

The size of GLUT4 clusters (D_cluster_∼100 nm) appears to be directly related to the diameter of the incoming GLUT4 vesicles (D_GSV_∼50 nm) and may reflect the possibility that the whole membrane of GSV flattens into the PM and forms a cluster. Membrane material inserted into the PM during GSV fusion may contain the necessary protein complexes for the formation of picket fence and retention of GLUT4. In this case the area of newly formed cluster would be equal to the area of the flattened GSV (πD^2^_GSV_ = ¼πD^2^_cluster_ = >D_cluster_ = 2D_GSV_ ∼ 100 nm). Further the observed elongated shapes of the clusters may be the result of fusion of tubular-vesicular GSV reported in the original EM studies [28].

GLUT4 clustering due to accumulation of GLUT4 at clathrin-coated pits is less likely, as the majority of clusters do not colocalize with clathrin or other components of endocytic machinery like AP2 and FCHo2 [11]. Analysis of time-resolved fluorescent signals for clathrin and GLUT4 also showed that conventional clathrin pits do not trigger GLUT4 clustering. Rather, clathrin is recruited to pre-existing GLUT4 clusters to mediate endocytosis of clathrin-coated vesicles enriched with GLUT4. Although we cannot exclude the possibility that another GLUT4-specific adaptor protein mediates GLUT4 clustering, our data argues against direct GLUT4 tethering or binding to a rigid structure associated with cytoskeleton, as clustered GLUT4 molecules did not immobilize, but appeared to move randomly within the borders of the cluster.

While this study can offer only indirect information about the mechanism of GLUT4 clustering, our data does exclude certain mechanisms of cluster formation. One commonly accepted model that we can rule out is the lipid raft [29], or the “liquid-ordered phase,” model. This model, which features condensed areas of the plasma membrane, could explain certain elements of GLUT4’s clustering behavior. Past literature proposed that GSV could transiently associate with lipid rafts [30], [31]. However, according to our data, the liquid-ordered lipid domain model is not likely because most clusters deviated significantly from a circular shape (circularity ∼0.34±0.15). Based on this circularity, it can be estimated that line tension for GLUT4 cluster should be less than 0.05 pN [32]. In contrast, line tension measured for a variety of sphingolipid-cholesterol enriched domains in phosphatidylcholine membranes of different acyl chain length, was always in the range of 0.8–6 pN, as estimated by hydrophobic mismatch calculations [32], [33]. Further, if the mechanism of GLUT4 clustering was dependent on cholesterol-enriched lipid domains, we would have expected that depletion of cholesterol would diminish the number of clusters [34]. However, when we depleted cholesterol we did not see disruption of the GLUT4 clusters. Both this lack of cholesterol-dependence and the elliptical shape of GLUT4 clusters are inconsistent with the lipid raft model.

Another popular model is the “picket fence,” or cytoskeleton mesh, generally used to account for diffusion of membrane molecules that exhibit transient confinement and deviation from random walk motion [35]. This model does not involve linear tension; rather the properties of confinement zones or “corrals” are solely governed by cytoskeleton meshwork, and can be of elongated and non-round shape [36]. The characteristic size of the transient confinement zones described in previous studies (∼190 nm) [26], [27] corresponds closely with typical cluster sizes we observed. However, the “picket fence” model proposes that the entire plasma membrane is divided into confinement zones by cytoskeleton meshwork, and would predict that molecules escaping one confinement zone would transition immediately into another [37]. In fact, this is very different from long-range free diffusion we observed for GLUT4 that dissociate from the cluster.

We propose instead a modification of the picket fence model, which can explain both a sparse density of GLUT4 clusters, and free diffusion of GLUT4 monomers between clusters. The picket fence model conceives of cytoskeleton meshwork attached to the inside of the PM. Some moving proteins are immobilized upon contact with this sub-membrane meshwork, and effectively become posts in a “picket fence” along the membrane skeleton. These corrals become transient confinement zones that block free diffusion throughout the cell. Our data forces us to extend this hypothesis by attributing the activity of protein selectivity to the corrals bound to the cytoskeleton meshwork, such that they cannot restrain all proteins. The clusters described in this paper would have a complement of GLUT4-specific ‘posts’, to account for the selective confinement of GLUT4 but not IRAP. These selective proteins would reflect GLUT4 but allow other proteins to pass, by recognizing common protein motifs.

This protein-specific fence model also accounts for the free diffusion of GLUT4 outside the clusters, as a relatively low density of GLUT4-specific confinement zones are required to fit our data. While not in these zones, the molecules would freely diffuse throughout the PM until reaching another cluster site. Since there is no line tension associated with this model, it can readily account for the elongated shapes of the observed clusters, assuming that several adjacent cytoskeleton meshwork sections can be joined into a single cluster. Indeed the characteristic size of the clusters estimated by FPALM and confirmed by EM corresponds very well to the length of one or two cytoskeleton meshwork sections. This organization of the membrane into protein-specific confinement zones may govern the dynamics of other PM proteins as well; a recent paper provides evidence of similar clustering behavior for the voltage-gated potassium channel Kv2.1 in PM [38]. They report selective confinement of Kv2.1, but not Kv1.4, in specialized clusters upon exocytosis.

Similar models have been proposed to govern spatial distribution and PM clustering of a number of other transmembrane and membrane-tethered proteins that can interact with cytoskeleton meshwork directly via actin-binding motifs, or indirectly by coupling to actin-binding proteins [39]. In contradistinction to our observations with GLUT4, however, the nanocluster distribution of glycosylphosphatidylinositol-anchored proteins is sensitive to perturbations of the cortical actin cytoskeleton, and its actomyosin contractility [40]. Conversely, nanoclusters of glycosylphosphatidylinositol-anchored proteins have been shown to fragment into monomers if the membrane has detached from the cytoskeleton, while the GLUT4 clusters, once formed, seem to be independent of the actin cytoskeleton. While these few experiments cannot fully rule out the role of actin in the formation and maintenance of hypothesized picket fences structures, our current inability to find any evidence for a role of actin does raise the possibility that molecules other than actin can act in different systems to form and maintain fences for the restricted diffusion of membrane proteins. The identities and mechanisms of these putative molecules involved in GLUT4 sequestration and organization in the clusters remain an open question for future studies.

### Conclusion

In response to insulin, GLUT4 storage vesicles fuse to the plasma membrane to increase cell glucose transport. Single molecule studies of the movement of GLUT4 molecules, genetically tagged with EOS, show a dynamic redistribution of GLUT4 monomers with clusters of GLUT4 (60–240 nm) containing high concentrations of GLUT4 that exhibit confined diffusion and increased residency time. GLUT4 dissociation from clusters into PM and endocytic uptake are directly affected by insulin and work in synergy with the increased delivery of monomers by exocytosis to maximize GLUT4 retention in PM for efficient transport of glucose. It remains to be seen if these new parameters are altered in disease states such as insulin resistance and type II diabetes.

## Materials and Methods

### Reagents

DMEM, Insulin, Alexa-488- and Alexa-594-conjugated secondary antibodies were all purchased from Invitrogen. Bovine serum albumin fraction V was from Intergen. Mouse anti-HA antibody (HA.11) was from Berkeley Antibody Co. (Richmond, CA). To generate HA-GLUT4-EOS, the tdEOS was amplified from pcDNA3_F1_EosFP (T158H/V123T) (cDNA kindly provided by Mike Davidson) using the primers 5′GCTTGGTACCATGGACTAC and GCTAGGATCCTTATCGTCTGG receiving Kpn1 (upstream) and BamHI site (downstream). The PCR product was ligated into digested HA-GLUT4-GFP pQB125 [22] to generate HA-GLUT4-EOS. The sequence of HA-GLUT4-EOS was verified by sequencing (MTR Scientific).

### Isolation and Transfection of Adipose Cells

Preparation of rat adipose cells, isolation and transfection were performed as described previously [41]. All procedures were performed according to the protocols approved by Institutional Animal Care and Use Committee of NIDDK (approval number K027-DB-10). All plasmids were used at a final concentration of 8 µg/ml. Transfected cells were kept in culture overnight and achieved optimal expression level at 20–24 h after electroporation. For imaging, cells were transferred to KRBH buffer with 1% BSA, pH 7.4, and maintained at 37°C in Delta-T environmental optical chamber (Bioptechs). Insulin stimulation was performed by addition of 70 nM insulin for 30 min at 37°C.

### TIRF and FPALM Imaging

FPALM setup was built around an inverted microscope (Nikon Ti) equipped with a TIRF-illumination arm, custom-built laser combiner (405, 488, 561 and 640 nm, Coherent), and an Andor Ixon EMCCD camera. A 60×1.49 NA objective was used for through-the-objective TIRF and wide-field illumination modes. The incident angle of the laser beam was controlled by a motorized TIRF-unit and switched between pre-calibrated settings corresponding to TIRF, and wide-field illumination. Penetration depth of the evanescent field was measured to be 110±10 nm by a calibration procedure with 40-nm fluorescent beads attached to the piezo-driven micropipette. Fluorescence signals generated during acquisition were separated from the excitation light using quad-band dichroic and emission filter set (405/488/561/640, Semrock). Sequential photo-activation and simultaneous time-lapse imaging of activated and non-activated HA-GLUT4-EOS was implemented using custom acquisition protocol in Micro-Manager 1.3.

### Single Molecule Tracking and Diffusion Analysis

Single-particle tracking and MSD were calculated with a modified version of the Particle Tracker PlugIn for ImageJ [42], using minimum and maximum threshold criteria to exclude spurious detections caused by camera noise and particles with intensity significantly brighter than single EOS molecules. Thresholds were experimentally determined from single molecule bleaching data (Fig. 1d). Intensity fluctuations caused loss of occasional particles; tracking was continued if it reappeared within three frames. We typically tracked 30–100 molecules per cell, and analyzed trajectories that were at least 30 frames long. For homogeneous two-dimensional diffusion, mean square displacement (MSD) is linearly related to time and can be determined from equation: MSD(t) = 4Dt+C, where D is the diffusion coefficient and C is the offset associated with precision of localization and instrumentation noise. Trajectories exhibiting minimal deviation from random diffusion were averaged together to estimate the diffusion coefficient. This diffusion coefficient (D_avg_ = 0.09 µm^2^/s) seeded simulations to determine 95% confidence intervals for individual realizations of random walk motion [43]. Based on these simulation data, all the remaining trajectories were classified as: free diffusion if MSD(t) was within the confidence intervals; and confined diffusion or directed motion if MSD(t) was outside of the confidence interval for at least five consecutive time points (Fig. 2d).

We used the FPALM PlugIn [44] to reconstruct positions of individual molecules within the clusters. Clusters were defined as diffraction-limited regions where GLUT4-EOS molecules were resident for more than 30 frames, i.e. significantly longer than could be expected for the diffusion-defined dwell time within the point-spread function. Reconstructed images were filtered to remove individual positions associated with free diffusion outside of the clusters. The resulting images were then analyzed with respect to the shape of the clusters. Characteristic width and length of the clusters was estimated by fitting with ellipses and measuring diameters along major and minor axis. Additionally, we used circularity measurement (4πA/P^2^, where A is the area of the cluster, and P is the perimeter) to account for clusters that significantly deviated from circular or elliptical shape.

Analysis of fusion and fission events was carried out using localization and detection of transient change of fluorescent signal in the IRAP-pHluorin or Clathrin-GFP channels as described previously [11]. The identified events were analyzed for associated change in GLUT4-EOS fluorescence. Unless otherwise stated, all data are represented as means ± SEM. Statistical significance was analyzed using Student’s t-test or ANOVA.

## Supporting Information

Figure S1 **Intracellular localization and insulin-induced translocation of HA-GLUT4-EOS to the plasma membrane detected by immunofluorescent microscopy.** (**a**) Isolated rat adipose cells transfected with HA-GLUT4-EOS were fixed, permeabilized and stained with mouse anti-HA (green) and rabbit anti-GLUT4(red) antibodies. Localization of HA and GLUT4 antibodies was visualized with corresponding secondary antibodies conjugated with Alexa-488 and Alexa-647. Under permeabilized conditions, HA and GLUT4 antibodies stained both intracellular and surface-exposed GLUT4. (**b**) Isolated rat adipose cells transfected with either HA-GLUT4-EOS or HA-GLUT4-GFP were fixed and stained with HA-antibody under non-permeabilized conditions. The HA-antibody labeled GLUT4 that was exposed at the cell surface, and was detected with a secondary antibody conjugated to Alexa-647. Total fluorescence of HA-antibody at the cell surface was averaged for 20 basal and 20 insulin-stimulated cells (30 min, 100 nM insulin at 37C). Data shown are means ± SEM. **p<0.01.
(TIF)

Figure S2 **GLUT4 clusters are insensitive to cholesterol depletion.** (**a**) Isolated rat adipose cells expressing HA-GLUT4-EOS were either kept in the basal condition or stimulated with insulin and then treated with 0.1 and 1 mM methyl-β-cyclodextrin (15 min at 37C). The cells were then fixed and stained with HA-antibody under non-permeabilized conditions. HA-antibody was detected with a secondary Alexa-647-conjugated antibody using TIRF illumination with a 640 nm laser. Individual diffraction-limited fluorescent structures were segmented and their density was measured as the number of structures per square micron for 30 cells for each condition. Data shown are means ± SEM. Depletion of cholesterol using 0.1–1 mM methyl-beta-cyclodextrin did not produce statistically significant changes in cluster density. (**b**) The effect of cholesterol depletion was also assessed on mobility of GLUT4 in the plasma membrane. Cells were treated with 1 mM methyl-β-cyclodextrin for 15 min at 37C and trajectories of single GLUT4-EOS molecules were acquired using FPALM. Mean Square Displacement (MSD) was calculated for trajectories of freely diffusing GLUT4-EOS molecules (MSD>2 um^2^). Graph shown is the average MSD ± SEM for 10 GLUT4 molecules from 3 different cells. The diffusion coefficient was estimated from the linear fit (red line) of the data (D_MbCD_ = 0.097±0.005 µm^2^/s) and was found to be similar to that of control cells (D_control_ = 0.092±0.008 µm^2^/s).
(TIF)

Figure S3 **Stability of GLUT4 clusters is independent of the actin cytoskeleton.** Isolated rat adipose cells expressing HA-GLUT4-EOS were treated for 15 min with cytochalasin D (1 uM) or latrunculin A (1 uM) before or after insulin stimulation. The cells were then fixed and stained with HA-antibody under non-permeabilized conditions and HA-antibody was detected with a secondary Alexa-647-conjugated antibody using TIRF illumination with 640 nm laser. Individual diffraction-limited fluorescent structures were segmented and their density was measured as the number of structures per square micron for 30 cells for each condition. Data shown are means ± SEM. Neither actin-disrupting drug produced statistically significant changes in cluster density when drugs were applied at basal or insulin-steady states. P-values for pair-wise comparison for basal state are: control vs. Lat A: p = 0.27; control vs. Cyt D: p = 0.1; for insulin-stimulated state (drug added after stimulation): control vs. Lat A: p = 0.44 and control vs. Cyt D: p = 0.33; for insulin-stimulated state (drug added before stimulation): control vs. Lat A: p<0.01; control vs. Cyt D: p = 0.3.* statistically different from corresponding control value, p<0.01, assessed by one-way ANOVA.
(TIF)

Movie S1 **Activation and random walk motion of HA-GLUT4-EOS over the whole area of the PM, without regions of exclusion.** To monitor single GLUT4 molecule diffusion simultaneously with bulk distribution of GLUT4 in PM, cells were co-transfected with HA-GLUT4-EOS and HA-GLUT4-GFP. Green channel shows conventional TIRF image of HA-GLUT4-GFP+non-activated HA-GLUT4-EOS. Red channel shows sparsely activated HA-GLUT4-EOS molecules. Scale bar, 5 µm.
(AVI)

Movie S2 **A GLUT4-EOS molecule exhibiting directed motion along a microtubule.** Cells were co-transfected with HA-GLUT4-EOS and tubulin-GFP and visualized using multi-color TIRF/FPALM. Green channel shows conventional TIRF image of tubulin-GFP. Red channel shows HA-GLUT4-EOS molecules activated in the wide-field photoactivation mode. Scale bar, 2 µm.
(AVI)

Movie S3 **A GLUT4 molecule undergoing rand om walk motion, observed using TIRF/FPALM microscopy of the HA-GLUT4-EOS probe. Scale bar, 1 µm.**
(AVI)

Movie S4 **A GLUT4 molecule undergoing confined diffusion within GLUT4 cluster, observed using TIRF/FPALM microscopy of the HA-GLUT4-EOS probe. Scale bar, 1 µm.**
(AVI)

Movie S5 **A GLUT4 molecule undergoing random walk motion, indicated by white arrow, is trapped and confined at the cluster, indicated by white circle.** To monitor single GLUT4 molecule diffusion simultaneously with bulk distribution of GLUT4 in PM, cells were co-transfected with HA-GLUT4-EOS and HA-GLUT4-GFP. Green channel shows conventional TIRF image of HA-GLUT4-GFP+non-activated HA-GLUT4-EOS. Red channel shows sparsely activated HA-GLUT4-EOS molecules. Scale bar, 2 µm.
(AVI)

Movie S6 **A GLUT4 molecule is released from its confined state within the cluster, indicated by white circle and moves away via random walk motion.** To monitor single GLUT4 molecule diffusion simultaneously with bulk distribution of GLUT4 in PM, cells were co-transfected with HA-GLUT4-EOS and HA-GLUT4-GFP. Green channel shows conventional TIRF image of HA-GLUT4-GFP+non-activated HA-GLUT4-EOS. Red channel shows sparsely activated HA-GLUT4-EOS molecules. Scale bar, 2 µm.
(AVI)

Movie S7 **Example of “fusion-with-dispersal” - GLUT4 vesicle fusion resulting in complete dispersal of GLUT4 molecules.** Cells were co-transfected with HA-GLUT4-EOS and IRAP-pHluorin and imaged 24 h after transfection. To monitor post-fusion dispersal of individual GLUT4 molecules, cells were first illuminated with 15–20 consecutive pulses of 405 nm laser at a wide-field illumination mode to achieve near complete photoconversion of HA-GLUT4-EOS into red-fluorescent state in PM and intracellular structures. Then cells were illuminated with a 561 nm laser in TIRF mode to selectively bleach HA-GLUT4-EOS molecules present at the PM. Red channel corresponds to activated HA-GLUT4-EOS molecules; green channel corresponds to Irap-pHluorin fluorescence. Irap-pHluorin fluorescence spike corresponds to luminal pH equilibration upon fusion pore opening. Site of fusion is indicated by white circle. Scale bar, 2 µm.
(AVI)

Movie S8 **Example of “fusion-with-retention” - GLUT4 vesicle fusion resulting in selective retention of GLUT4 molecules and cluster formation at the site of the fusion.** Cells were co-transfected with HA-GLUT4-EOS and IRAP-pHluorin and imaged 24 h after transfection. To monitor post-fusion dispersal of individual GLUT4 molecules, cells were first illuminated with 15–20 consecutive pulses of 405 nm laser at a wide-field illumination mode to achieve near complete photoconversion of HA-GLUT4-EOS into red-fluorescent state in PM and intracellular structures. Then cells were illuminated with a 561 nm laser in TIRF mode to selectively bleach HA-GLUT4-EOS molecules present at the PM. Red channel corresponds to activated HA-GLUT4-EOS molecules; green channel corresponds to Irap-pHluorin fluorescence. Irap-pHluorin fluorescence spike corresponds to luminal pH equilibration upon fusion pore opening. Scale bar, 2 µm.
(AVI)
