# Supplementary figures and images for: Insulin Regulates Glut4 Confinement in Plasma Membrane Clusters in Adipose Cells
Source: PLoS One. 2013 Mar 8;8(3):e57559. doi: 10.1371/journal.pone.0057559 (PMC3592853; doi:10.1371/journal.pone.0057559)

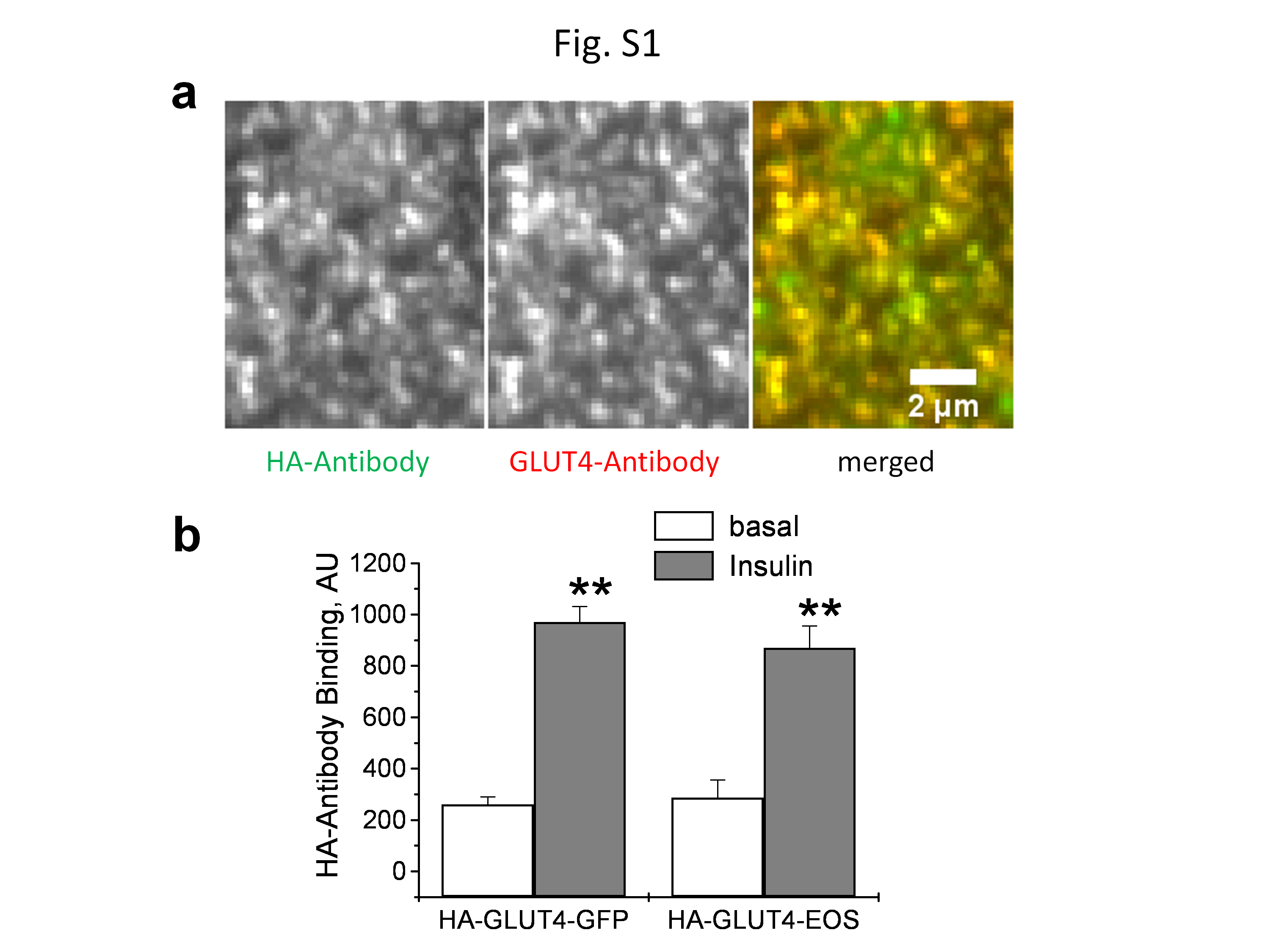

Supplement: Figure S1 — Intracellular localization and insulin-induced translocation of HA-GLUT4-EOS to the plasma membrane detected by immunofluorescent microscopy. (a) Isolated rat adipose cells transfected with HA-GLUT4-EOS were fixed, permeabilized and stained with mouse anti-HA (green) and rabbit anti-GLUT4(red) antibodies. Localization of HA and GLUT4 antibodies was visualized with corresponding secondary antibodies conjugated with Alexa-488 and Alexa-647. Under permeabilized conditions, HA and GLUT4 antibodies stained both intracellular and surface-exposed GLUT4. (b) Isolated rat adipose cells transfected with either HA-GLUT4-EOS or HA-GLUT4-GFP were fixed and stained with HA-antibody under non-permeabilized conditions. The HA-antibody labeled GLUT4 that was exposed at the cell surface, and was detected with a secondary antibody conjugated to Alexa-647. Total fluorescence of HA-antibody at the cell surface was averaged for 20 basal and 20 insulin-stimulated cells (30 min, 100 nM insulin at 37C). Data shown are means ± SEM. **p<0.01. (TIF) [file pone.0057559.s001.tif]

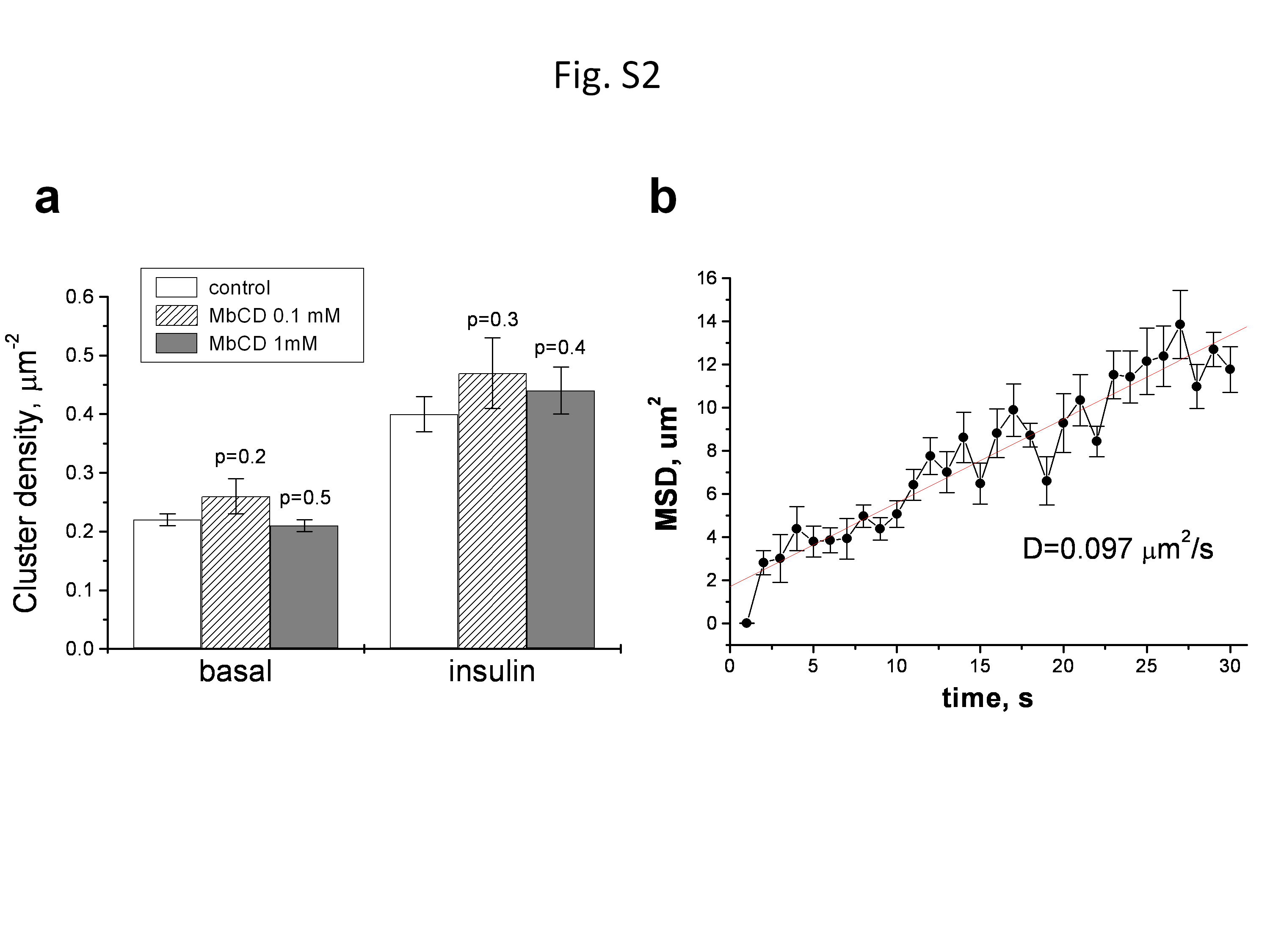

Supplement: Figure S2 — GLUT4 clusters are insensitive to cholesterol depletion. (a) Isolated rat adipose cells expressing HA-GLUT4-EOS were either kept in the basal condition or stimulated with insulin and then treated with 0.1 and 1 mM methyl-β-cyclodextrin (15 min at 37C). The cells were then fixed and stained with HA-antibody under non-permeabilized conditions. HA-antibody was detected with a secondary Alexa-647-conjugated antibody using TIRF illumination with a 640 nm laser. Individual diffraction-limited fluorescent structures were segmented and their density was measured as the number of structures per square micron for 30 cells for each condition. Data shown are means ± SEM. Depletion of cholesterol using 0.1–1 mM methyl-beta-cyclodextrin did not produce statistically significant changes in cluster density. (b) The effect of cholesterol depletion was also assessed on mobility of GLUT4 in the plasma membrane. Cells were treated with 1 mM methyl-β-cyclodextrin for 15 min at 37C and trajectories of single GLUT4-EOS molecules were acquired using FPALM. Mean Square Displacement (MSD) was calculated for trajectories of freely diffusing GLUT4-EOS molecules (MSD>2 um2). Graph shown is the average MSD ± SEM for 10 GLUT4 molecules from 3 different cells. The diffusion coefficient was estimated from the linear fit (red line) of the data (DMbCD = 0.097±0.005 µm2/s) and was found to be similar to that of control cells (Dcontrol = 0.092±0.008 µm2/s). (TIF) [file pone.0057559.s002.tif]

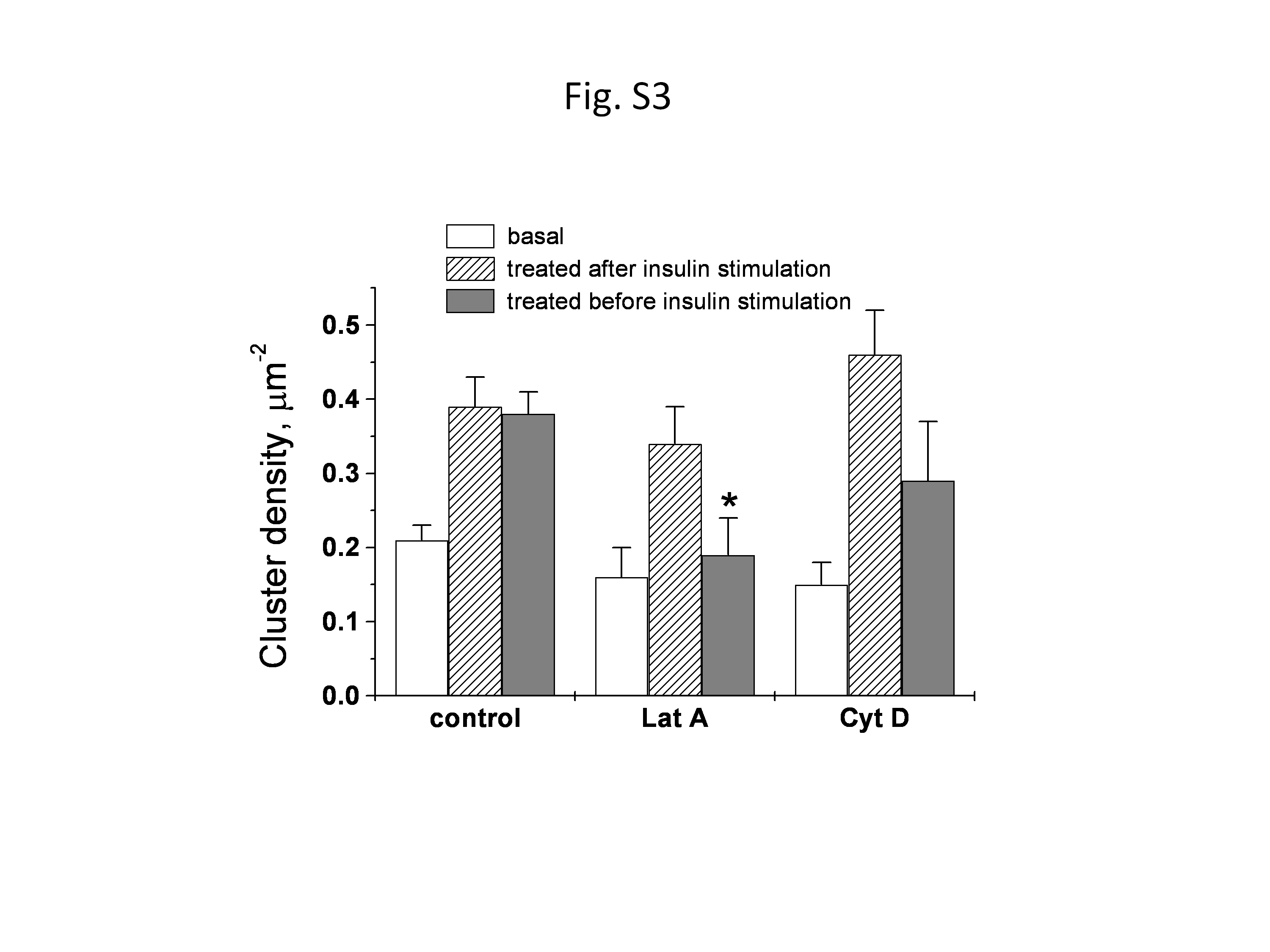

Supplement: Figure S3 — Stability of GLUT4 clusters is independent of the actin cytoskeleton. Isolated rat adipose cells expressing HA-GLUT4-EOS were treated for 15 min with cytochalasin D (1 uM) or latrunculin A (1 uM) before or after insulin stimulation. The cells were then fixed and stained with HA-antibody under non-permeabilized conditions and HA-antibody was detected with a secondary Alexa-647-conjugated antibody using TIRF illumination with 640 nm laser. Individual diffraction-limited fluorescent structures were segmented and their density was measured as the number of structures per square micron for 30 cells for each condition. Data shown are means ± SEM. Neither actin-disrupting drug produced statistically significant changes in cluster density when drugs were applied at basal or insulin-steady states. P-values for pair-wise comparison for basal state are: control vs. Lat A: p = 0.27; control vs. Cyt D: p = 0.1; for insulin-stimulated state (drug added after stimulation): control vs. Lat A: p = 0.44 and control vs. Cyt D: p = 0.33; for insulin-stimulated state (drug added before stimulation): control vs. Lat A: p<0.01; control vs. Cyt D: p = 0.3.* statistically different from corresponding control value, p<0.01, assessed by one-way ANOVA. (TIF) [file pone.0057559.s003.tif]
